# Natural Language Processing for Depression Prediction on Sina Weibo: Method Study and Analysis

**DOI:** 10.2196/58259

**Published:** 2024-09-04

**Authors:** Zhenwen Zhang, Jianghong Zhu, Zhihua Guo, Yu Zhang, Zepeng Li, Bin Hu

**Affiliations:** 1 Gansu Provincial Key Laboratory of Wearable Computing, School of Information Science and Engineering, Lanzhou University, Lanzhou, China

**Keywords:** depression, social media, natural language processing, deep learning, mental health, statistical analysis, linguistic analysis, Sina Weibo, risk prediction, mood analysis

## Abstract

**Background:**

Depression represents a pressing global public health concern, impacting the physical and mental well-being of hundreds of millions worldwide. Notwithstanding advances in clinical practice, an alarming number of individuals at risk for depression continue to face significant barriers to timely diagnosis and effective treatment, thereby exacerbating a burgeoning social health crisis.

**Objective:**

This study seeks to develop a novel online depression risk detection method using natural language processing technology to identify individuals at risk of depression on the Chinese social media platform Sina Weibo.

**Methods:**

First, we collected approximately 527,333 posts publicly shared over 1 year from 1600 individuals with depression and 1600 individuals without depression on the Sina Weibo platform. We then developed a hierarchical transformer network for learning user-level semantic representations, which consists of 3 primary components: a word-level encoder, a post-level encoder, and a semantic aggregation encoder. The word-level encoder learns semantic embeddings from individual posts, while the post-level encoder explores features in user post sequences. The semantic aggregation encoder aggregates post sequence semantics to generate a user-level semantic representation that can be classified as depressed or nondepressed. Next, a classifier is employed to predict the risk of depression. Finally, we conducted statistical and linguistic analyses of the post content from individuals with and without depression using the Chinese Linguistic Inquiry and Word Count.

**Results:**

We divided the original data set into training, validation, and test sets. The training set consisted of 1000 individuals with depression and 1000 individuals without depression. Similarly, each validation and test set comprised 600 users, with 300 individuals from both cohorts (depression and nondepression). Our method achieved an accuracy of 84.62%, precision of 84.43%, recall of 84.50%, and *F*_1_-score of 84.32% on the test set without employing sampling techniques. However, by applying our proposed retrieval-based sampling strategy, we observed significant improvements in performance: an accuracy of 95.46%, precision of 95.30%, recall of 95.70%, and *F*_1_-score of 95.43%. These outstanding results clearly demonstrate the effectiveness and superiority of our proposed depression risk detection model and retrieval-based sampling technique. This breakthrough provides new insights for large-scale depression detection through social media. Through language behavior analysis, we discovered that individuals with depression are more likely to use negation words (the value of “swear” is 0.001253). This may indicate the presence of negative emotions, rejection, doubt, disagreement, or aversion in individuals with depression. Additionally, our analysis revealed that individuals with depression tend to use negative emotional vocabulary in their expressions (“NegEmo”: 0.022306; “Anx”: 0.003829; “Anger”: 0.004327; “Sad”: 0.005740), which may reflect their internal negative emotions and psychological state. This frequent use of negative vocabulary could be a way for individuals with depression to express negative feelings toward life, themselves, or their surrounding environment.

**Conclusions:**

The research results indicate the feasibility and effectiveness of using deep learning methods to detect the risk of depression. These findings provide insights into the potential for large-scale, automated, and noninvasive prediction of depression among online social media users.

## Introduction

### Background

Depression is a global mental illness that is affecting the physical and mental health of an increasing number of people worldwide. In recent years, despite the World Health Organization and national governments introducing relevant policies for the diagnosis and treatment of depression, the significant challenge remains in early detection and timely treatment for a larger number of potential patients with depression [1,2]. Researchers have been exploring the potential application of clinical assessments [3,4], biological markers [5-9], and imaging techniques [10-12] in detecting depression, but there is still a lack of widely accepted and validated objective biological markers or imaging techniques for clinical diagnosis. Therefore, the diagnosis of clinical depression still heavily relies on clinical assessments and subjective symptom reports. The rapid proliferation of mobile internet technology has encouraged more individuals to share their lives and emotions on social media platforms. Meanwhile, the accumulation of vast amounts of user-generated content has sparked researchers’ interest in studying the mental health of social media users within the academic community [13-16].

### Challenges

Early studies primarily relied on feature-based statistical methods to learn the differences between individuals with depression and those without. Several statistical features, such as emotional words [17], language style [18], and social behavior [19] were widely used. Although these features played a crucial role in studying the differences between depressed and nondepressed groups at the time, they did not support more in-depth research and further exploration. Additionally, due to the limitations of early data collection technologies, conclusions drawn from small-scale data sets may not generalize well to larger user populations. With the rapid development of natural language processing (NLP) and deep learning, many scholars have explored applying these technologies to depression detection tasks on social media [20]. Some popular neural network models, such as convolutional neural networks (CNNs) and recurrent neural networks are widely used to encode user posts to obtain a user-level semantic representation [21-26].

Existing research treats depression detection as a long text classification task, where user posts are concatenated into a long text and then encoded through neural networks. However, these methods face several significant challenges. (1) The concatenated long text loses the fine-grained emotional information expressed in different posts and faces challenges in terms of computational speed and computing resources. (2) The existing research uses all collected user posts to train the model, which is worth discussing. Not all posts from a user express symptoms, emotions, or thoughts related to depression. (3) Previous studies have mainly focused on English social media, and the findings of these studies lack adaptability and generalizability to Chinese social media.

### Contributions

To address the above challenges, we first constructed a depression detection data set based on Sina Weibo, containing 527,333 posts from 1600 users with depression and 1600 users without depression. We propose a hierarchical transformer network (HTN) model to obtain a high-quality user-level semantic representation. The model mainly consists of a 2-level transformer structure that focuses on learning semantic representations at the post level and the user level. For each user, the model first uses a transformer encoder to encode each post and obtain post-level semantic representations. Then, these post embeddings are further encoded by another transformer encoder and aggregated through a long short-term memory (LSTM) with attention to obtaining user-level semantic representations. This structure not only effectively considers the sequential evolutionary relationships of user emotional changes but also dynamically evaluates the importance of different posts. In addition, we also propose a retrieval-based post sampling strategy to mitigate the impact of noise on the model training process. Specifically, we construct a depression-related dictionary to match user posts with relevant content for model training. Experimental results demonstrate that the model and sampling strategy proposed in this paper achieve promising results on the constructed depression detection data set. This fully illustrates the sophistication and effectiveness of the proposed model and sampling strategy. Our methodology provides strong support for identifying users at risk of depression through online social media data in Chinese communities, which is important for public health and social harmony.

Our contributions can be summarized as follows: (1) we propose a hierarchical transformer-based model that can effectively capture both local and global semantic information from user posts; (2) we propose a retrieval-based post sampling strategy that effectively reduces noise in user post data and improves the quality of user-level semantic representations; and (3) we construct a depression detection data set consisting of 3200 online social media users, with over 527,333 posts collected from 1600 users with depression and 1600 users without depression.

### Related Works

With the rapid growth of mobile social media, an increasing number of people are sharing their daily lives and emotional states online. As a result, researchers have become interested in using artificial intelligence technology to detect mental health issues, particularly depression, from social media data [27]. Early studies, however, were limited by small data sets and the development of NLP. These studies primarily focused on detecting depression using feature-based statistical methods, examining features such as emotional words, social engagement, and language style [19,22,28]. Researchers also explored the use of depressive-related expressions on Twitter, finding that individuals with depression tend to use more negative language in their online posts compared to those without depression [23]. Additionally, they established an evaluation task using NLP to identify individuals with depression and posttraumatic stress disorder on social media by building a data set of approximately 1800 individuals from Twitter [24]. Furthermore, they investigated the linguistic disparities between individuals with and individuals without depression by analyzing discussions of depression-related topics on social media platforms.

With the advent of deep learning and neural network technologies, there has been a significant breakthrough in detecting depression through social media. These technologies have enhanced feature extraction capabilities, allowing for the automatic capture of complex semantic information from user-generated content. They excel in semantic understanding and sentiment analysis, particularly in accurately identifying users’ emotional states using attention mechanisms and recurrent neural networks [10]. They used machine learning methods to analyze photos from 166 Instagram users, suggesting that color analysis, metadata components, and algorithmic facial detection may serve as effective markers for detecting depression in photos [14]. They built a depression data set based on Reddit self-reported depression diagnosis(RSDD) and suggested using CNN to learn embedded representations for each post [17]. They proposed an integrated multi-classifier depression detection method, revealing the effectiveness of ensemble learning on depression detection tasks [26], constructed a depression detection data set based on Twitter, and proposed a method that integrates multiple semantic representations to detect depressive individuals [29]. They introduced a collaborative representation model based on reinforcement learning, which automatically selects depression-related posts and images from user-generated data to enhance depression detection performance [30]. They proposed an attention-based feature fusion model, which achieved good predictive performance on small-scale data sets [31], and a multimodal depression recognition framework that combines deep convolutional networks (DCNNs) and deep neural networks (DNNs). DCNNs are used to learn local feature representations for each modality, while DNNs integrate various features for final prediction [32]. They integrated tweet and user behavioral features, encoding user tweets using a hierarchical attention network [33], and investigated the depression classification capability of 3 bidirectional encoder representation from transformer (BERT) variants and 4 combinations of BERT variants on the text responses to 12 clinical interview questions. They found that ensemble methods could improve both *F*_1_-scores and robustness [34] and proposed a multimodal fusion method for depression detection, where BERT is used to obtain the sentence representation and LSTM and CNN are employed to capture the representation of speech.

Although previous studies have explored the detection of depression using social media from the perspectives of features and encoding models and achieved significant results, there are still some issues that need to be further investigated [35-37]. User-level depression detection faces 2 key issues. First is the design of neural semantic encoders that balance performance and computational speed. Second is the quality control of user posts. Specifically, previous work has treated the classification of users with depression as a long-text classification task. User posts are concatenated into long text for encoding, which not only loses the emotional or sentiment information expressed in different posts but also creates a text length that is difficult to adapt to models like Transformer [38] and BERT [39]. It is worth noting that, despite BERT’s remarkable performance improvement in many NLP tasks, it relies on pretraining knowledge from large-scale general domains. However, this general domain knowledge does not match well with the specific domain knowledge of depression. Additionally, more computational resources are strongly required in scenarios based on the BERT model. Therefore, this poses greater challenges for applying BERT to user sequence modeling.

## Methods

### Data Collection and Annotation

**Figure 1.**
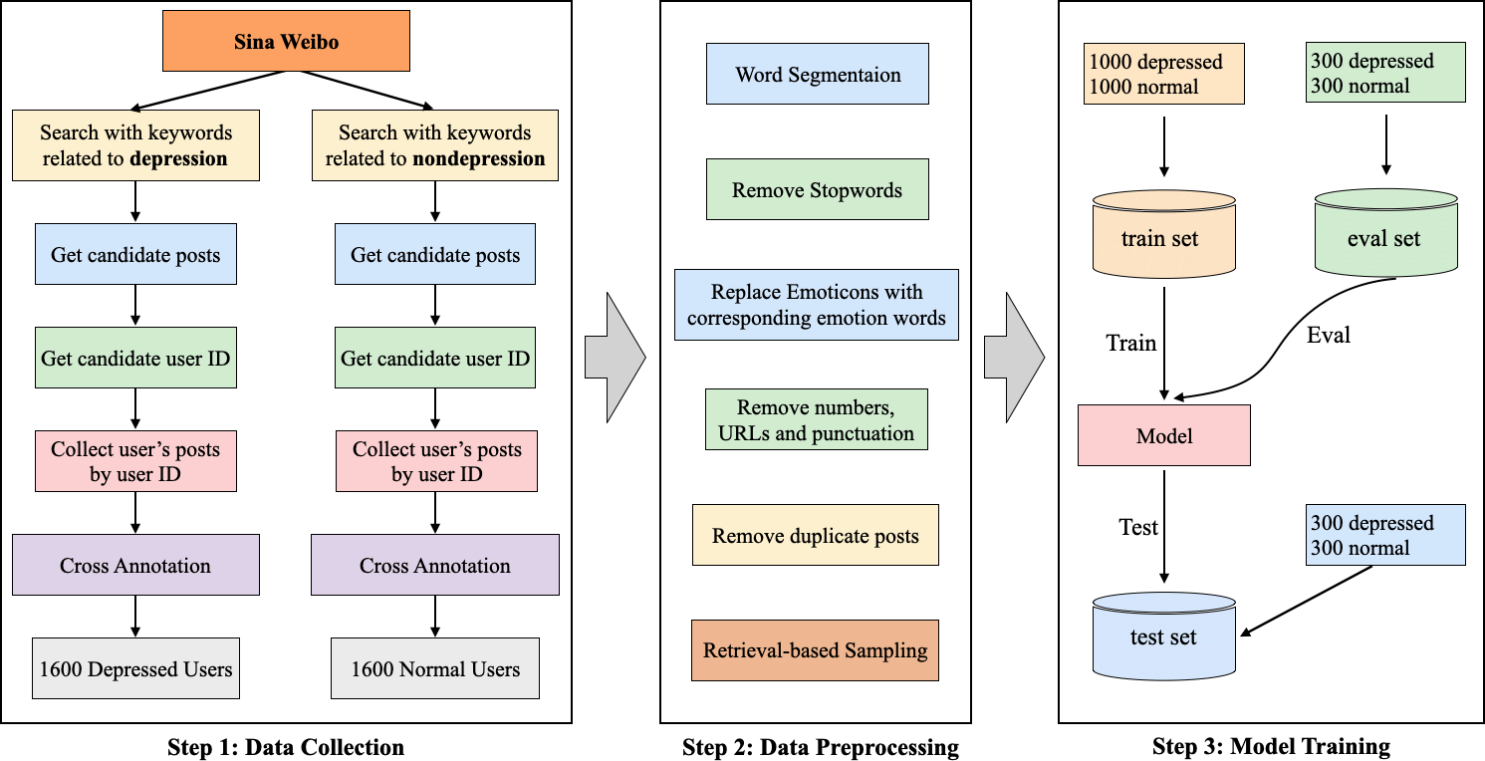
The workflow for data set construction and model learning. Eval set: evaluation set; Train set: training set.

Figure 1 illustrates the workflow of constructing a user-level depression detection data set based on the Sina Weibo platform, which includes 3 steps: data collection, data preprocessing, and model training. In the following sections, we provide a detailed explanation and description of these steps.

### Step 1: Data Collection

#### Overview

We followed the annotation guidelines [29,32] proposed in studies on English social media for depression. If a user self-reported in their post that they were diagnosed with depression, then we annotated the user as depressed. For nondepressed users, if the posts they published did not clearly reveal symptoms or keywords related to depression, we annotated them as nondepression users (normal users). The detailed process is given in the sections below.

#### Search With Keywords

We employed 2 methods for retrieval. One method involved directly searching for “depression” as a keyword on Weibo. The other method involved using keywords such as “depression,” “symptoms,” and “medication names” within the depression supertopic on Sina Weibo.

#### Get Candidate Posts

We manually selected posts from individuals genuinely experiencing depression and removed posts related to popular science.

#### Get Candidate User ID

We obtained user IDs of candidate posts through the Weibo platform’s field parsing system.

#### Crawl User’s Posts by User ID

We used web crawling technology to scrape posts published by users on the Sina Weibo platform within a specific time period.

#### Cross-Annotation

Three annotators cross-annotated users based on the scraped posts, labeling them as depression or nondepression. When the decisions of the 3 annotators were consistent, we considered the user as valid and included them in either the depression or nondepression group. The determination principle for depression users was as follows: if a user voluntarily reported being diagnosed with depression in their posts, we labeled them as having depression. Additionally, we also considered expressions in the Chinese context, such as mentioning medication or suicidal thoughts. The determination principle for nondepression users was that their posts did not explicitly contain expressions related to depression.

### Step 2: Data Preprocessing

The raw data collected from Sina Weibo often contains irrelevant or informal expressions, which can negatively impact the model’s performance. To address this issue, we processed the raw data using the following steps: (1) user-identifiable information was removed to protect user privacy; (2) each post was segmented into a word sequence using the Jieba tokenizer for efficient processing; (3) emoticons were replaced with their corresponding emotion words for more accurate analysis; (4) numbers, URLs, and punctuation were eliminated from the posts to reduce noise; (5) automatically generated posts by Sina Weibo’s robot assistant, such as birthday reminders and membership-level notifications, were filtered out; (6) duplicate posts were removed to ensure data uniqueness; and (7) posts consisting of fewer than 3 words were excluded from training to maintain quality standards.

### Step 3: Model Training

Since the data set we constructed was balanced, we divided the 1600 depression and nondepression users into training, validation, and testing sets, with 1000 users for training, 300 for validation, and 300 for testing. Therefore, a total of 2000 users were used for training, 600 users for validation, and 600 users for testing.

### Ethical Considerations

All data in this study were obtained from publicly shared information on Sina Weibo, and any personal information that could potentially expose user privacy was excluded from the study. Therefore, this analysis applied the standards for waiving informed consent and similar guidelines [40]. In addition, our research complied with the requirements of the Sina Weibo platform regarding the use of user data. We ensured that our study did not involve infringement of user privacy or ethical issues. Specifically, we desensitized and anonymized the collected user data, removing any information that could potentially indicate user identities during the preprocessing stage. Furthermore, since this study used a limited data set of Sina Weibo users for modeling and analysis, these conclusions may not fully generalize to all depression and nondepression users on Sina Weibo. The predictive outcomes of the model should be considered as suggested conclusions and not be regarded as definitive decisions in the real world.

### Problem Definition

This study aimed to develop a depression risk prediction model using NLP and user-generated data from social media. The input to this model was each user’s posts, and the output was a label indicating whether the user is depressed or not.

### Proposed Model

#### Overview

**Figure 2.**
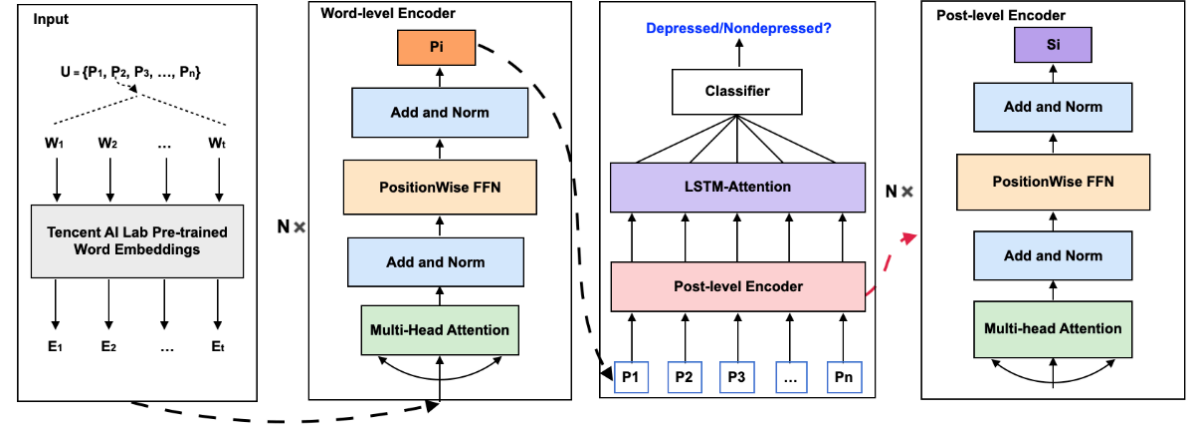
The architecture of our proposed depression prediction model. FFN: feedforward neural network; LSTM-Attention: long short-term memory with attention.

Figure 2 illustrates the workflow of our proposed depression detection model, which consists of 5 steps: word embedding, post embedding, user embedding, classification, model training, and evaluation. We provide detailed insights into the development and training in the following sections.

As shown in Figure 2, we propose an HTN to study textual semantic features from users’ posts. The Transformer is an attention-based neural network architecture that has gained considerable attention in recent years, particularly in NLP and computer vision. Unlike other deep learning models, the Transformer not only dynamically captures long-term dependencies but also exhibits faster computation speed. Inspired by this, we incorporated the Transformer into our model to better understand and encode behavior and intention from user posts. Our model consists of 2 levels of transformers: a word-level transformer and a post-level transformer. The word-level transformer is used to compute semantic features for each post, with word embeddings from each post as input. The sentence-level transformer is employed to calculate aggregated semantic features for all user posts, with the input being the embeddings of all user posts. After obtaining the aggregated global feature representation, we performed classification on it to predict whether the user is depressed. Since our prediction task is a binary classification task, we used a sigmoid function for prediction. The proposed model is capable of learning fine-grained feature representations at the levels of words, sentences, and documents from user posts, which is crucial for enhancing prediction accuracy.

#### Word Embedding

To obtain better word embeddings, we used Tencent’s pretrained word embeddings [41] (Tencent AI Lab Embedding Corpus for Chinese Words and Phrases) to initialize the embedding representations of each word in user posts. This embedding corpus was pretrained on Wikipedia, Baidu Baike, and web text data using the Directional Skip-Gram algorithm, and it includes embeddings for 12,287,936 Chinese words. Specifically, we first employed the vocabulary from the Tencent pretrained word embedding database as external vocabulary for tokenizing each user post with the Jieba tokenizer. Then, we retrieved the embedding for each word in the user posts from the Tencent pretrained word embedding database and input them into the model for further training.

#### Post Embedding

After obtaining the pretrained word embeddings, we added positional encodings to each word in the posts and combined them with the word embeddings. These new embeddings were then fed into the first-level transformer encoder for encoding, where each transformer encoder consists of a multi-head self-attention mechanism and a feed-forward neural network. The self-attention mechanism allows each word to interact with other words in the sequence, while the feed-forward neural network applies independent nonlinear transformations to each word. Each sublayer uses residual connections and layer normalization to stabilize the training process. After processing through multiple layers, the contextual representation of each word is obtained, with the representation of the [CLS] token being used as the final embedding representation of the post.

#### User Embedding

As described above, we employed a shared transformer encoder to obtain semantic embeddings for each post. To effectively merge these post embeddings, we employed another transformer encoder along with an LSTM network equipped with an attention mechanism for deeper semantic feature extraction and aggregation of each user’s posts. Specifically, the embeddings of user posts are sequentially input into the transformer encoder in the order of their posting time for deep feature extraction. Subsequently, the semantic context obtained from the transformer encoder is processed by an attention-based LSTM structure to extract and aggregate sequential information. The advantage of this model architecture is that it not only learns more effective deep semantic contextual representations but also dynamically considers the importance of different posts.

#### Classification

We focused on predicting whether a user is at risk of depression, thus a binary classification process was applied to the user embeddings.

#### Model Training and Evaluation

We divided the raw data into 3 sets: the training set, validation set, and test set. The training set consists of 1000 depressed and 1000 nondepressed users, the validation set consists of 300 depressed and 300 nondepressed users, and the test set consists of 300 depressed and 300 nondepressed users. All models were implemented using the PyTorch [42] framework on a graphics processing unit (GPU) server equipped with 2 Tesla A100 cards. For the CNN model, the convolutional kernel size was set to {2, 3, 4}, and the number of filters was set to 100. For other baselines, both the hidden size and attention size were set to 256. For our proposed model, each post was padded or truncated to 512 words. The learning rate was set to 1e-3, and the batch size was optimized from the range of {32, 64, 128}.

### Comparison Baselines

To comprehensively evaluate the potential of applying deep learning for predicting depression risk on social media, we adopted 11 widely used neural network models as baselines. These included CNN, LSTM, gated recurrent unit (GRU), bidirectional GRU, and bidirectional LSTM models and attention-based methods like LSTM with attention, GRU with attention, bidirectional LSTM with attention, and bidirectional GRU with attention, BERT, and a hierarchical convolutional network model.

### Evaluation Metrics

We used accuracy, macroaveraged precision, macroaveraged recall, and macroaveraged F1-score to evaluate the models presented in this study. These metrics are widely used to assess the performance of deep learning–based models.

## Results

### Performance Comparison

Table 1 presents the experimental results of the baseline models and our proposed model on the test set. We observed that our proposed model achieves over 80% accuracy in predicting depression risk across all scenarios. Compared with neural models without the attention mechanism, attention-based neural models demonstrate better detection performance across all sampling strategies, with particularly significant improvements observed when using the no-sampling strategy. We attribute this improvement to the attention mechanism’s ability to automatically focus more on words or phrases indicative of depression, thereby facilitating a superior semantic representation of the user. The HTN model outperforms the other baseline models, with at least a 2% improvement in the retrieval strategy and more than a 5% improvement in the other conditions. This suggests that encoding a user’s post data with HTN is more effective than treating it as a single long text. HTN enables the model to fully consider post interactions and intuitively fit better with human thinking. Simply treating all of a user’s posts as a single long text may lead to computational and gradient challenges, limiting the model’s ability to detect depression.

**Table 1.** Overall performance comparison of our proposed model and baseline models. Without: results without applying any sampling strategy; Random: results of applying random sampling strategy to sample 50% of posts; Retrieval: results based on retrieval sampling strategy.

Model		Accuracy	Precision	Recall	*F*_1_-score
**CNN** ^**a**^					
	Without	79.93	80.70	80.79	79.93
	Random	78.37	78.30	78.63	78.29
	Retrieval	93.53	93.21	93.54	93.30
**LSTM** ^**b**^					
	Without	71.80	73.71	69.91	69.91
	Random	69.55	69.35	68.52	68.65
	Retrieval	88.41	88.40	88.10	88.23
**GRU** ^**c**^					
	Without	78.55	78.98	77.55	77.86
	Random	77.68	78.03	76.69	76.98
	Retrieval	92.25	92.09	92.49	92.21
**BiGRU** ^**d**^					
	Without	67.99	67.77	67.94	67.81
	Random	70.24	70.02	69.30	69.43
	Retrieval	91.52	91.35	91.63	91.46
**BiLSTM** ^**e**^					
	Without	65.92	65.88	66.06	65.81
	Random	65.05	65.19	65.36	64.99
	Retrieval	84.95	84.95	85.35	84.90
**LSTM-attention** ^**f**^					
	Without	78.55	78.34	78.15	78.23
	Random	74.05	73.77	73.90	73.82
	Retrieval	91.87	91.72	92.13	91.82
**GRU-attention** ^**g**^					
	Without	82.53	82.43	82.12	82.24
	Random	80.62	80.39	80.43	80.41
	Retrieval	91.27	91.16	91.34	91.15
**BiLSTM-attention** ^**h**^					
	Without	78.03	77.94	77.42	77.59
	Random	74.39	74.20	73.72	73.87
	Retrieval	91.35	91.58	90.05	91.19
**BiGRU-attention** ^**i**^					
	Without	80.97	80.75	80.97	80.83
	Random	76.64	76.72	77.03	76.59
	Retrieval	92.77	92.68	92.88	92.64
**BERT** ^**j**^					
	Without	81.44	80.37	80.52	80.11
	Random	79.92	78.42	78.66	78.21
	Retrieval	90.21	89.48	88.71	89.05
**HCN** ^**k**^					
	Without	83.33	83.19	83.84	83.41
	Random	78.62	80.66	79.39	79.77
	Retrieval	93.53	93.34	94.02	93.40
**HTN** ^**l**^					
	Without	84.62	84.43	84.50	84.32
	Random	82.43	82.24	82.44	82.35
	Retrieval	95.46	95.30	95.70	95.43

a CNN: convolutional neural network.

b LSTM: long short-term memory.

c GRU: gated recurrent unit.

d BiGRU: bidirectional gated recurrent unit.

e BiLSTM: bidirectional long short-term memory.

f LSTM-attention: long short-term memory with attention.

g GRU-attention: gated recurrent unit with attention.

h BiLSTM-attention: bidirectional long short-term memory with attention.

i BiGRU-attention: bidirectional gated recurrent unit with attention.

j BERT: bidirectional encoder representation from transformer.

k HCN: hierarchical convolutional network.

l HTN: hierarchical transformer network; (best performing model).

### Effectiveness of Sampling Strategy

**Figure 3.**
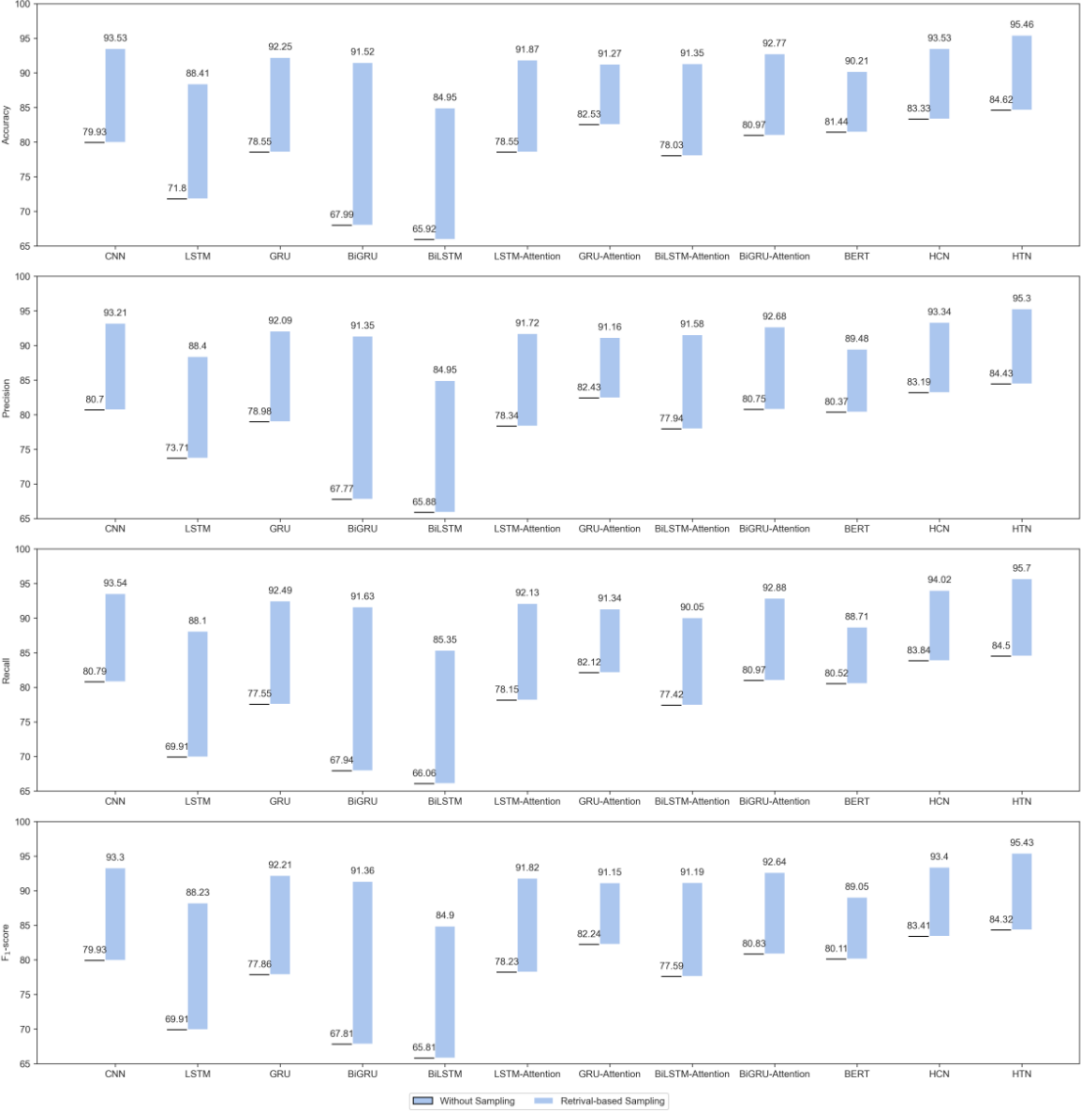
Performance comparison between applying retrieval-based sampling strategy and not applying any sampling strategy. BERT: bidirectional encoder representation from transformer; BiGRU: bidirectional gated recurrent unit; BiGRU-attention: bidirectional gated recurrent unit with attention; BiLSTM: bidirectional long short-term memory; BiLSTM-attention: bidirectional long short-term memory with attention; CNN: convolutional neural network; GRU: gated recurrent unit; GRU-Attention: gated recurrent unit with attention; HCN: hierarchical convolutional network; HTN: hierarchical transformer network; LSTM: long short-term memory; LSTM-Attention: long short-term memory with attention.

Figure 3 illustrates the comparison of model performance before and after applying our proposed retrieval-based sampling strategy. After applying the retrieval-based sampling strategy, the proposed model’s depression risk prediction accuracy exceeds 95%. These fully highlight the necessity and importance of sampling user posts. Through sampling, the computational overhead of model training can be effectively reduced, allowing the model to focus more on learning about depression. In addition, we also noticed that the random sampling strategy performed worse than the no-sampling strategy, likely due to the inherent uncertainty in the random sampling process.

**Figure 4.**
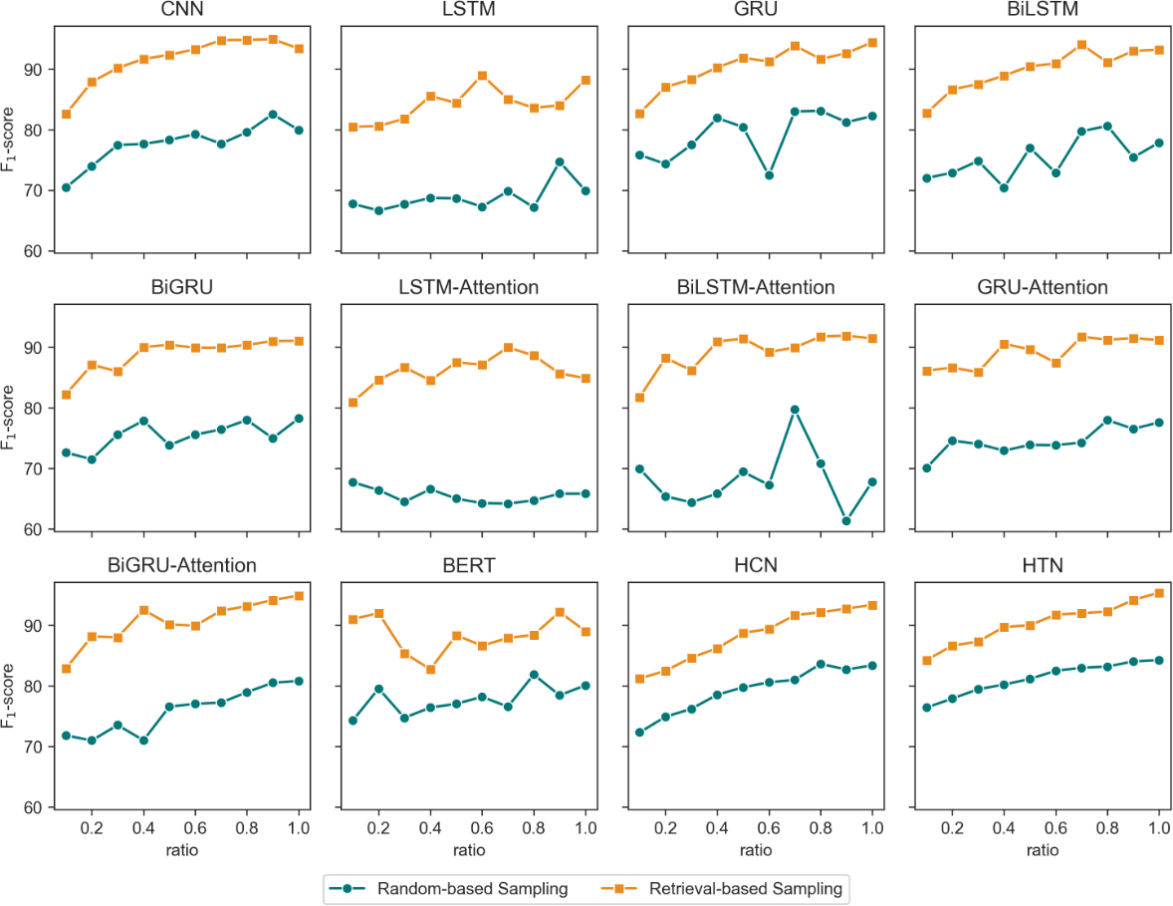
Comparison of model performance results with different sampling strategies and sampling ratios. BERT: bidirectional encoder representation from transformer; BiGRU: bidirectional gated recurrent unit; BiGRU-attention: bidirectional gated recurrent unit with attention; BiLSTM: bidirectional long short-term memory; BiLSTM-attention: bidirectional long short-term memory with attention; CNN: convolutional neural network; GRU: gated recurrent unit; GRU-Attention: gated recurrent unit with attention; HCN: hierarchical convolutional network; HTN: hierarchical transformer network; LSTM: long short-term memory; LSTM-Attention: long short-term memory with attention.

Figure 4 illustrates the *F*_1_-scores of each model under various sampling strategies and sampling ratios. It is evident that the application of effective sampling strategies can significantly enhance the depression detection capabilities of the models. Conversely, in random sampling experiments, achieving performance beyond that of the full data set (sampling rate of 1.0) is challenging when the sampling rate is less than 1.0. By employing a retrieval-based sampling strategy to select posts relevant to depression, not only is the computational complexity of the model reduced, but the model also gains a better focus on acquiring knowledge related to depression from user posts. We observed that the retrieval-based sampling strategy consistently demonstrated a stable upward trend as the sampling rate increased incrementally, unlike the random sampling strategy, which exhibited more pronounced fluctuations. We attribute this primarily to the fact that the retrieval-based sampling strategy ensures the selection of posts related to depression in each sampling iteration. Conversely, the post selection process in the random sampling strategy is probabilistic and does not guarantee the relevance of a user’s post to depression in each selection.

### Linguistic and Behavior Analysis

**Figure 5.**
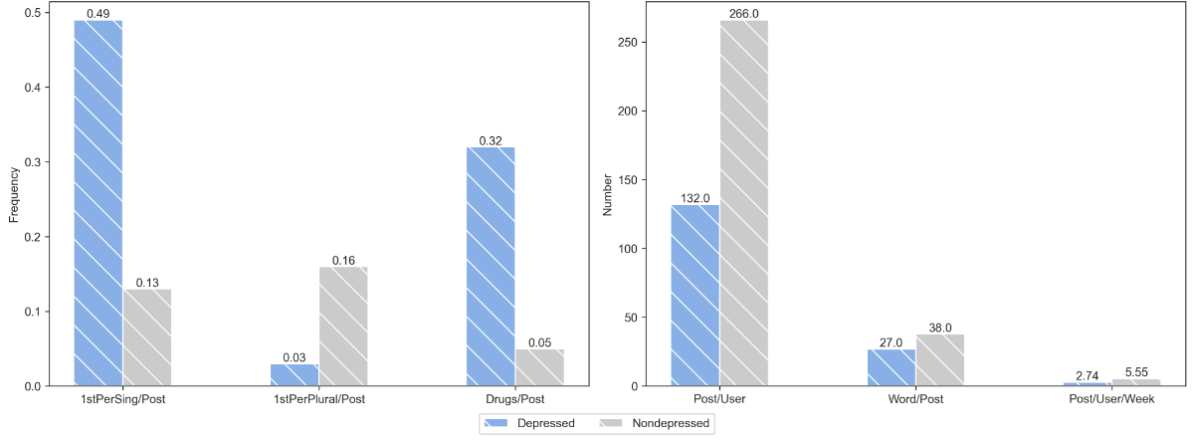
Comparison of the social behaviors between depressed and nondepressed users. “Word/Post”: the average number of words per post; “Post/User”: the average number of posts per user; “Post/User/Week”: the average number of posts per user per week; “1stPerSing/Post”: the frequency of the first-person singular (我) used per post; “1stPerPlural/Post”: the frequency of the first-person singular (我们) used per post; “depression/Post”: the frequency of the keywords (抑郁症, 抑郁) used per post; “Drugs/Post”: the frequency of mentioning depression medication–related terms per post.

Figure 5 compares the common differences in social behaviors between depressed and nondepressed users. We can observe that, compared with nondepressed users, depressed users have fewer posts and lower posting frequency, reflecting the less active social engagement of depressed users. In terms of pronoun use, depressed users tend to use the first-person singular (我) more frequently in their posts, while nondepressed users use the first-person plural (我们) more often. This suggests that depressed users may be more self-focused and have less interaction with others, whereas nondepressed users are more group-oriented and engage in more interactive behaviors. Additionally, depressed users are more likely to focus on depression-related topics on social media, such as discussing their condition, treatment processes, and medication, while nondepressed users mention and discuss these topics less frequently.

**Figure 6.**
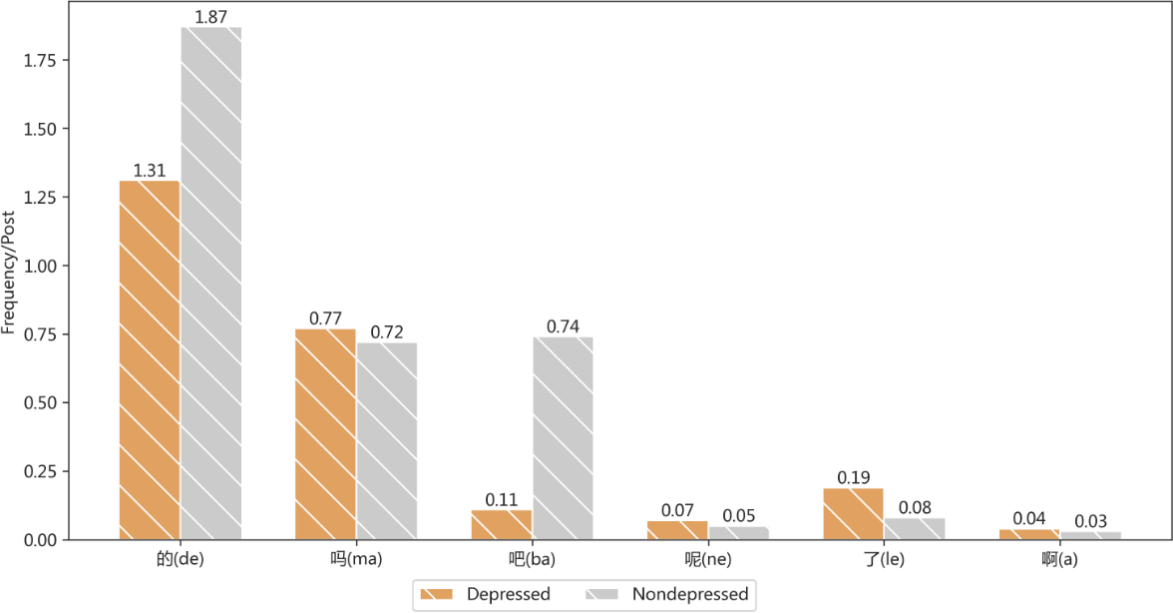
Comparison of the modal particle use between depressed and nondepressed users.

Figure 6 presents the comparative results of modal particle use between depressed users and nondepressed users. We can observe that the use of “的” (de) is more frequent in both depressed and nondepressed users, while “呢” (ne) is used the least frequently. The main reason is that “的” is commonly used as a modifier in almost all sentences, whereas “呢” and “吗” are often used in contexts expressing questions or uncertainties. It is worth noting that “吧” (ba) is used more frequently in the language expressions of users with depression, while “啊” (a) is used more frequently in the language expressions of nondepressed users. These 2 words are typically used at the end of sentences, with “吧” often used to modify completed events, while “啊” is typically used to modify events that are about to happen. In the expressions of users with depression, “吧” is more often expressed as “好吧” (“okay”), “行吧” (“all right”), “就这样吧” (“just like this”), “去死吧” (“go die,”), etc. On the other hand, “啊” is often combined in expressions of nondepressed users as “真开心啊” (“really happy”), “原来是这样啊” (“so that’s how it is”), and “你对我真好啊” (“you’re really good to me”).

**Figure 7.**
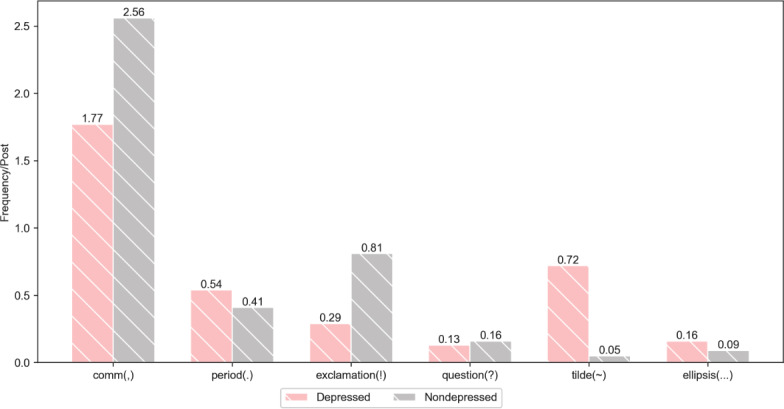
Comparison of the punctuation use between depressed and nondepressed users.

Figure 7 illustrates the comparative results of punctuation use between depressed users and nondepressed users. We discovered that depressed users tend to use periods more frequently than nondepressed users, while nondepressed users prefer commas over those with depression. We speculate that this trend may stem from the fact that depressed users often experience low moods and slowed thinking, which could manifest in more cautious and negative expressions. A period can signify a conclusion or a clear break between ideas, possibly reflecting the psychological inclination of these individuals to conclude or avoid further communication. In contrast, nondepressed users typically exhibit active and divergent thinking patterns. They frequently employ commas to separate sentence components and convey incomplete thought processes.

Additionally, we observed that nondepressed users are more inclined to use exclamation marks (“!”), which aligns with the experimental results regarding the interjection “啊” (“a”) presented in Figure 6. Furthermore, depressed users tend to use the tilde (“~”) and ellipses more frequently. These symbols are commonly employed in the Chinese internet context to convey a sense of helplessness or resignation.

**Figure 8.**
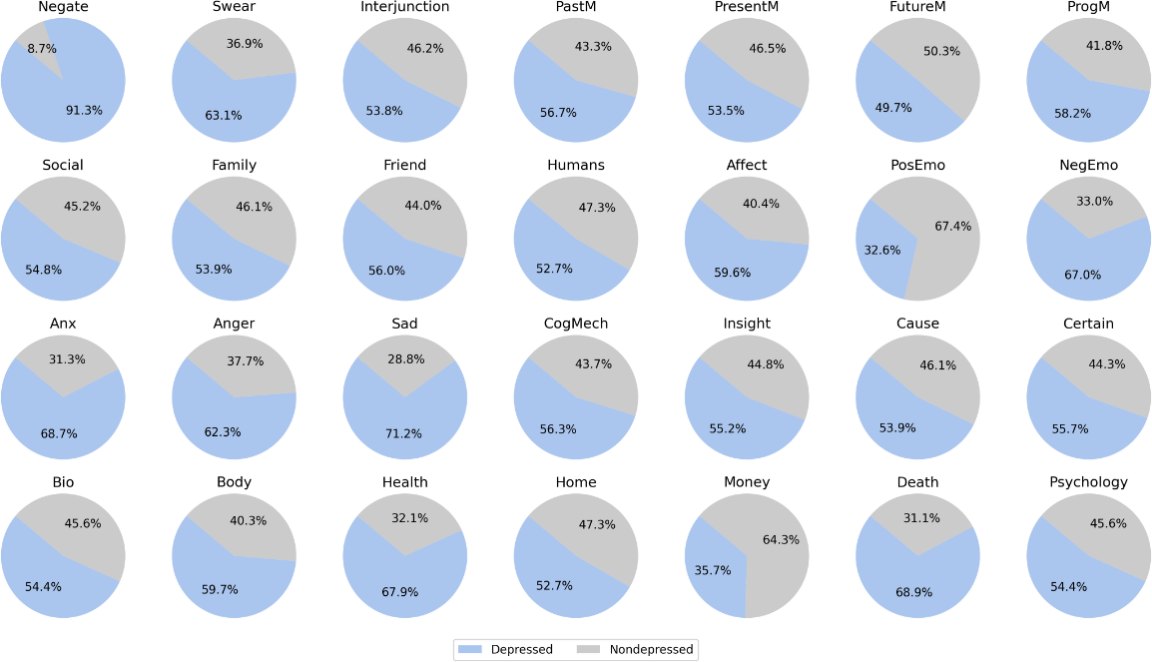
Comparison of significant Linguistic Inquiry and Word Count features between depressed and nondepressed users.

We used the Chinese Linguistic Inquiry and Word Count (LIWC) dictionary [43] to analyze the differences in language use between users with depression and nondepressed users, and Figure 8 presents the comparative results. Figure 8 reveals that users with depression are more likely to use negative vocabulary, such as “Swear,” “Affect,” “PosEmo,” “NegEmo,” “Anx,” “Anger,” “Sad,” etc, than nondepressed users. Depressed users appeared to favor discussing past and present events (“PastM,” “PresentM”), whereas nondepressed users appeared to focus more on possible future events (“FutureM”). We speculated that this difference might be attributed to the significant influence of their family of origin on many depressed users, leading them to reflect more on the impact of past events in their posts. Furthermore, we observed that depressed users exhibited relatively more negative than nondepressed users when discussing topics related to “Social,” “Family,” “Friends,” and “Home.” Additionally, we found that words such as “Bio,” “Body,” “Health,” “Death,” and “Psychology” were more frequently used in the posts of depressed users. The main reason for this is that posts by depressed users may express their intentions related to suicide or self-harm, or they may involve sharing experiences and discussions about the condition among fellow patients, encompassing the diagnosis process, physical condition, and medication.

## Discussion

### Principal Results

This paper explores the automatic prediction of depression risk among users on online social media using deep learning methods. We developed and validated the model on a large-scale data set of online social media users. The research findings indicate that the proposed model exhibits significant advantages in predicting depression risk, confirming the effectiveness and advanced capabilities of deep learning for depression risk prediction. The paper carries several implications.

With the rapid development of social media technology, more and more young people are using social media to share their emotions and document their lives. Social media has become a vital platform for them to express emotions, seek support, and build social connections. However, mental health issues among young people are increasingly prominent, making them a key societal concern. Social media serves as a vital tool for them to communicate their feelings and connect with others. However, it also poses a challenge in effectively using social media data to identify and support individuals who may be facing mental health issues. More and more individuals with mental health problems, especially depression, do not actively seek help from professionals. This leads to a lack of timely treatment and support, causing them to miss optimal intervention opportunities. Furthermore, there is a growing shortage of clinical psychologists to meet the increasing mental health needs of the population. Therefore, exploring automated depression risk identification technologies based on artificial intelligence, particularly deep learning, has become a crucial and essential research topic in addressing the current societal challenges.

Furthermore, this study developed an HTN and proposed a retrieval-enhanced post sampling technique to improve the performance of depression risk detection. Experimental results indicate that our developed approach outperforms all baseline methods, achieving prediction accuracies and *F*_1_-scores of 84% across 3 independent experiments. With the application of the retrieval sampling technique, the performance of almost all methods reaches nearly 90%. Compared with methods without sampling, there is a performance improvement of over 10% across all 4 metrics. This strongly demonstrates the effectiveness and advanced capabilities of our approach in predicting the risk of depression.

Finally, linguistic analysis revealed that depressed users exhibit more conservative and reserved social behaviors on social media compared with nondepressed users. Not only do they make fewer posts, but their posts are also shorter. This may reflect their negativity in social interactions and a tendency to avoid social engagement. Reduced social engagement could result from the loneliness, frustration, or lack of motivation commonly felt by individuals with depression. Additionally, depressed users express more negative emotions in their posts. Through linguistic sentiment analysis, we found that posts by depressed users contain more negative sentiment words, a difference more pronounced than in nondepressed users. This further highlights the psychological distress and negative emotional experiences that individuals with depression may encounter on social media. These traits offer insights into the behaviors of depressed users, providing direction for developing more accurate and personalized depression risk prediction models.

### Limitations

Although our research has achieved some promising results, there are still some limitations. These limitations mainly focus on the 3 aspects given below.

#### Research Data

This study relies on a subset of users from the Chinese social media platform Sina Weibo, which may not fully represent the Chinese population or all users of Chinese social media. Considering the individual differences among users, the research model and results of this study may not accurately assess the depression risk of internet users. Additionally, the findings of this study may not be generalizable to users of other social media platforms or populations with different medical conditions.

#### Chinese LIWC

A notable limitation is that the existing Chinese LIWC dictionary covers a limited vocabulary. It may not fully capture all the emotional and semantic nuances in the texts of depressed and nondepressed users, especially as language and culture evolve and new expressions emerge, which the dictionary might not update to include in a timely manner. Another limitation is that LIWC mainly analyzes based on word frequency and lacks contextual understanding. It cannot discern the different meanings of polysemous words in various contexts, nor can it handle complex grammatical structures and sentence-level emotional expressions. Additionally, LIWC focuses on surface-level vocabulary analysis and lacks the ability to comprehend deep semantics and implied meanings. It cannot effectively handle sarcasm, metaphors, and complex emotional expressions. Therefore, when using LIWC for text analysis, we should combine it with other methods and tools to obtain more comprehensive and accurate results. We also need to remain critical of LIWC’s output and consider its limitations when interpreting research conclusions.

#### Large Language Model

Although large language models demonstrate powerful capabilities in semantic representation, we did not explore this in our paper. Our main concerns regarding this are as follows. First, they demand high computational resources, including a large number of GPU or tensor processing unit resources as well as significant storage space. Second, due to their large number of parameters, they require longer training times, which may incur substantial time and cost. Additionally, the complexity of large language models poses a risk of overfitting, necessitating additional regularization and tuning. Furthermore, large language models have poor interpretability, making it difficult to understand and explain their internal structure and decision-making processes. Last, large language models require a large amount of training data, which may raise concerns about the use and protection of user privacy data, necessitating additional data management and security measures.

### Conclusions

In this study, we explored using deep learning techniques to predict depression risk based on social media data. We collected posts from 3200 online social media users over a 1-year period in order to develop and validate a depression risk detection model. The proposed HTN demonstrated exceptional performance on the collected data, yielding a predictive accuracy of over 95% across 4 commonly employed evaluation metrics. Furthermore, we introduced a retrieval-based post sampling technique, which significantly improved our model’s ability to detect the risk of depression. This research provides technical support for the automatic identification of users at risk of depression on Chinese online social media, thereby effectively supporting online platforms in engaging in societal risk management.

## Abbreviations

BERT: bidirectional encoder representation from transformer
CNN: convolutional neural network
DCNN: deep convolutional network
DNN: deep neural network
GPU: graphics processing unit
GRU: gated recurrent unit
HTN: hierarchical transformer network
LIWC: Linguistic Inquiry and Word Count
LSTM: long short-term memory
NLP: natural language processing
